# The Antinociceptive, Antioxidant and Anti-Inflammatory Effects of 5-Fluoro-2-Oxindole during Inflammatory Pain

**DOI:** 10.3390/antiox9121249

**Published:** 2020-12-09

**Authors:** Alejandro Redondo, Gabriela Riego, Olga Pol

**Affiliations:** 1 Grup de Neurofarmacologia Molecular, Institut d’Investigació Biomèdica Sant Pau, Hospital de la Santa Creu i Sant Pau, 08041 Barcelona, Spain; josealejandro.redondo@e-campus.uab.cat (A.R.); gabrielaalejan.riego@e-campus.uab.cat (G.R.); 2 Grup de Neurofarmacologia Molecular, Institut de Neurociències, Universitat Autònoma de Barcelona, 08193 Barcelona, Spain

**Keywords:** analgesia, inflammation, morphine, opioids, oxidative stress, oxindole, pain

## Abstract

Recent studies demonstrate that 5-fluoro-2-oxindole inhibits neuropathic pain but the antinociceptive actions of this drug and its effects on the plasticity, oxidative and inflammatory changes induced by peripheral inflammation as well as on the effects and expression of µ-opioid receptors (MOR) have not been evaluated. In C57BL/6 male mice with inflammatory pain provoked by the subplantar administration of complete Freund’s adjuvant (CFA), we evaluated: (1) the antinociceptive actions of 5-fluoro-2-oxindole and its reversion with the HO-1 inhibitor, tin protoporphyrin IX (SnPP); (2) the effects of 5-fluoro-2-oxindole in the protein levels of mitogen-activated protein kinase (MAPK), Nrf2, NADPH quinone oxidoreductase1 (NQO1), heme oxygenase 1 (HO-1), oxidative stress marker (4-hydroxy-2-nonenal; 4-HNE), inducible nitric oxide synthase (NOS2), microglial markers (CD11b/c and IBA-1), and MOR in the spinal cord and/or paw of animals with inflammatory pain; (3) the antinociceptive effects of morphine in 5-fluoro-2-oxindole pre-treated animals. Treatment with 5 and 10 mg/kg of 5-fluoro-2-oxindole inhibited the allodynia and hyperalgesia induced by CFA in a different, time-dependent manner. These effects were reversed by SnPP. Treatment with 5-fluoro-2-oxindole increased the expression of NQO1, HO-1 and MOR and inhibited the CFA-induced upregulation of phosphorylated MAPK, 4-HNE, NOS2, CD11b/c and IBA-1 in spinal cords and/or paws. The local effects of morphine were improved with 5-fluoro-2-oxindole. This work reveals that 5-fluoro-2-oxindole inhibits the plasticity, oxidative and inflammatory responses provoked by peripheral inflammation and potentiates the antinociceptive effects of morphine. Thus, treatment with 5-fluoro-2-oxindole alone and/or combined with morphine are two remarkable new procedures for chronic inflammatory pain management.

## 1. Introduction

Several studies showed the protective role played by numerous oxindole derivates such as isorhynchophylline (IRN) and rhynchophylline (RIN), versus diabetes, thrombosis, bacterial infection, asthma, cancer and inflammation [1,2,3,4]. The antinociceptive actions of different oxindole alkaloids during acute inflammation or visceral pain have also been demonstrated [5,6,7]. A recent study further revealed the antinociceptive effects of 5-fluoro-2-oxindole in animals with chronic neuropathic pain [8]. Nonetheless, the potential pain-relieving action of 5-fluoro-2-oxindole in animals with chronic inflammatory pain has not yet been evaluated.

Microglial cells are decisive for the progress and preservation of inflammatory pain [9,10]. Microglial activation initiates the synthesis of inflammatory mediators, for instance tumor necrosis factor α (TNFα), several interleukins and inducible nitric oxide synthase (NOS2), which promote inflammatory pain. Peripheral inflammation also induced the activation of mitogen-activated protein kinase (MAPK)/NF-κB signaling pathways in the spinal cord and paw [11], whose inhibition is a mechanism of action of the oxindoles, RIN and IRN, for attenuating the inflammatory responses induced by lipopolysaccharide (LPS) in cell cultures [12,13]. The administration of 5-fluoro-2-oxindole also inhibits the spinal cord and hippocampal activation of microglia provoked by nerve injury [8]. Nonetheless, the regulatory effects of 5-fluoro-2-oxindole in MAPK phosphorylation, microglial activation and NOS2 overexpression in the spinal cord and/or paw tissues of mice with inflammatory pain have not been evaluated.

The antioxidant enzymes, NADPH quinone oxidoreductase1 (NQO1) and/or heme oxygenase 1 (HO-1), participate in the analgesic actions of several compounds such as sulforaphane, oltipraz and carbon monoxide-releasing molecules (CORM’s) during inflammatory and neuropathic pain [11,14,15,16,17]. Other works also proved that the analgesic effects of 5-fluoro-2-oxindole in animals with sciatic nerve injury-induced neuropathic pain are mediated via augmenting the Nrf2 transcription factor, NQO1 and HO-1 protein levels [8] and that the anti-inflammatory effects of IRN were produced via avoiding the down-regulation of the antioxidant proteins superoxide dismutase, glutathione peroxidase 1 and catalase [3]. The probable contribution of the Nrf2 transcription factor, NQO1 and HO-1 in the pain-relieving actions of 5-fluoro-2-oxindole, and the effects of this treatment in the levels of 4-hydroxy-2-nonenal (4-HNE)-positive proteins, an oxidative stress marker, during inflammatory pain were not identified.

Several works demonstrated that different drugs are able to potentiate the analgesic effects of µ-opioid receptor (MOR) agonists in animals with neuropathic or inflammatory pain. Thus, the effectiveness of morphine increased in animals with inflammatory or neuropathic pain pre-treated with activators of Nrf2 (sulforaphane) or HO-1 (cobalt protoporphyrin IX (CoPP)) as well as with CORM’s [11,14,15,18,19]. 5-fluoro-2-oxindole also enhanced the antiallodynic and antihyperalgesic effects of morphine in sciatic nerve-injured animals [8], but its role in modulating the analgesic properties of morphine and MOR expression during inflammatory pain remains untested.

In mice with chronic inflammatory pain generated with the subplantar injection of complete Freund’s adjuvant (CFA), we assessed the effects of 5-fluoro-2-oxindole in: (a) the mechanical allodynia and thermal hyperalgesia provoked by peripheral inflammation and the reversion of its effects with the administration of the HO-1 inhibitor, tin protoporphyrin IX (SnPP); (b) the protein levels of MAPK, Nrf2, HO-1 and NQO1, the oxidative stress (4-HNE) and inflammatory (NOS2, CD11b/c and IBA-1) markers as well as of MOR in the spinal cord and/or paw tissues; (c) the antinociceptive actions of locally administered morphine.

## 2. Materials and Methods

### 2.1. Animals

Male C57BL/6 mice (21–25 g), acquired at Envigo Laboratories (Barcelona, Spain), were accommodated under 12/12 h light/dark conditions in a room with controlled temperature of 22 °C and humidity of 66% until use. These animals with free access to food and water were used after 7 days acclimatization to the housing conditions. All the planned experiments were performed between 9:00 a.m. and 5:00 p.m. and carried out in conformity with the guidelines of the European Commission’s directive (2010/63/EC), the Spanish Law (RD 53/2013) regulating animal research and approved by the local Committee of Animal Use and Care of the Autonomous University of Barcelona (the ethical code is 1325R5). Maximal exertions to diminish animal suffering and the number of animals used were made.

### 2.2. Induction of Inflammatory Pain

Chronic inflammatory pain was incited with the subplantar injection of CFA (30 µL; Sigma-Aldrich, St. Louis, MO, USA) into the right hindpaw under brief anesthetic conditions with isoflurane in accordance with our previous work [20].

### 2.3. Mechanical Allodynia

Mechanical allodynia was evaluated by measuring the hindpaw withdrawal response to von Frey filament stimulation. Mice were sited in methacrylate cylinders (20 cm high × 9 cm in diameter; Servei Estació, Barcelona, Spain) on a wire grid bottom, across which von Frey filaments (North Coast Medical, Inc., San Jose, CA, USA) with bending force in the range 0.008–3.5 g were applied by using the up/down paradigm [21]. The test was imitated with the 0.4 g filament, and the strength of the following filament was increased or decreased according to the response. The threshold of response was calculated from the sequence of filament strength used during the up/down procedure using an Excel program (Microsoft Iberia SRL, Barcelona, Spain), which includes the adjustment to the data curve. A clear paw withdrawal, licking the paw, or shaking the paw was considered a nociceptive like response.

### 2.4. Thermal Hyperalgesia

Thermal hyperalgesia was evaluated by assessing the paw withdrawal latency in response to radiant heat in the plantar test (Ugo Basile, Varese, Italy) [22]. Animals were positioned in Plexiglas tubes (20 cm high × 9 cm diameter) placed on a glass surface. The heat source was situated under the plantar surface of the hind paws and activated with a light beam intensity until paw withdrawal. The cut-off time is 12 s. Paw withdrawal latencies were determined from the mean of three separate assays.

In both tests, animals were familiarized to the environment for 1 h before the test so that they were quiet. Both ipsilateral and contralateral hind paws were tested.

### 2.5. Western Blot Analysis

Naïve and CFA-injected mice treated with 5 mg/kg 5-fluoro-2-oxindole or vehicle for 11 days were euthanized by cervical dislocation at 0 and 14 days after CFA injection. The spinal cord of the lumbar section and the subplantar tissue of the hind legs of the ipsilateral side were extracted, frozen and kept at −80 °C until use. Protein levels of p-JNK/JNK, p-ERK 1/2/ERK 1/2, p-P38/P38, Nrf2, HO-1, NQO1, 4-HNE, NOS2, CD11b/c, IBA-1 and MOR, were analyzed. The homogenization of tissues was done in ice-cold lysis buffer (50 mM Tris·Base, 150 nM NaCl, 1% NP-40, 2 mM EDTA, 1 mM phenylmethylsulfonyl fluoride, 0.5 Triton X-100, 0.1% sodium dodecyl sulfate, 1 mM Na_3_VO_4_, 25 mM NaF, 0.5% protease inhibitor cocktail, and 1% phosphatase inhibitor cocktail). NP-40 was purchased from Calbiochem (Darmstadt, Germany) and all other reagents were acquired from Sigma-Aldrich. After solubilization of crude homogenate for 1 h at 4 °C, it was sonicated for 10 s and centrifuged at 4 °C for 15 min at 700× *g*. Then, the supernatant (60 µg of total protein) was mixed with 4× Laemmli loading buffer and loaded onto 4% stacking/10–12% separating sodium dodecyl sulfate polyacrylamide gels. After that, proteins were electrophoretically transferred onto a polyvinylidene fluoride membrane for 120 min and blocked with phosphate-buffered saline with Tween 20 plus 5% nonfat dry milk or Tris-buffered saline with Tween 20 plus 5% nonfat dry milk or 5% bovine serum albumin for 1 h and 15 min, and then incubated with specific rabbit primary antibody anti-phospho-JNK, total JNK, phospho-ERK 1/2, total ERK 1/2, and total P38 (1:250; Cell Signaling Technology, Danvers, MA, USA), and phospho-P38 (1:200; Cell Signaling Technology), Nrf2 (1:160; Abcam, Cambridge, UK), HO-1 (1:200; Abcam), NQO1 (1:333; Sigma-Aldrich), 4-HNE (1:150, Abcam), NOS2 (1:150; Abcam), CD11b/c (1:200; Novus Biologic, Littleton, CO, USA), IBA-1 (1:150; Thermo Fisher Scientific, Waltham, MA, USA) and (MOR (1:333; Merck, Billerica, MA, USA) overnight at 4 °C. A horseradish peroxidase-conjugated anti-rabbit secondary antibody (GE Healthcare, Little Chalfont, UK) was used to detect proteins, which were visualized with chemiluminescence reagents (ECL kit; GE Healthcare, Little Chalfont, UK) and exposure to Kodak film. Blot intensity was quantified by densitometry using Image-J program (National Institutes of Health, Bethesda, MD, USA). A rabbit anti-glyceraldehyde-3-phosphate dehydrogenase (GAPDH) antibody (1:5000; Merck, Billerica, MA, USA) was used as a loading control.

### 2.6. Experimental Procedures

At first, we investigated the mechanical antiallodynic and thermal antihyperalgesic effects of the daily intraperitoneal administration of 5 and 10 mg/kg of 5-fluoro-2-oxindole or vehicle (dimethylsulfoxide 1% solution in saline) from day 4 to 14 after CFA injection (*n* = 6 animals per group). We used contralateral paws as controls.

Therefore, we evaluated the reversion of the antiallodynic and antihyperalgesic effects generated by 5 mg/kg 5-fluoro-2-oxindole intraperitoneally administered during 11 consecutive days with the intraperitoneal administration of 5 mg/kg SnPP, a selective HO-1 inhibitor. SnPP was administered 30 min after 5-fluoro-2-oxindole injection and animals were tested 30 min after SnPP injection (*n* = 6 animals per group). The dose of SnPP was selected in accordance with other studies [18,23].

The protein levels of p-JNK/JNK, p-ERK 1/2/ERK 1/2, p-P38/P38, Nrf2, HO-1, NQO1, 4-HNE, NOS2, CD11b/c, IBA-1 and MOR in the ipsilateral site of the spinal cords and paws from mice with peripheral inflammation treated with 5 mg/kg 5-fluoro-2-oxindole or vehicle during 11 consecutive days were evaluated by Western blot assay. We used naive mice treated with vehicle as controls (*n* = 3–4 samples per group).

In other groups, we investigated the antiallodynic and antihyperalgesic actions induced by co-administration of 5-fluoro-2-oxindole (5 mg/kg, intraperitoneal) or vehicle with morphine (50 µg, subplantar) or saline during inflammatory pain. Mice were tested at 30 min after morphine administration (*n* = 6 animals per group). The dose of morphine was selected in accord with previous studies [11,19].

The researcher who executed these tests was not aware of the treatments used.

### 2.7. Drugs

**Figure 1:**
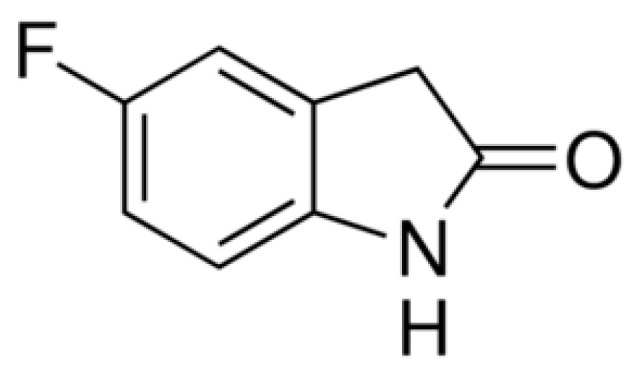
Chemical structure of 5-fluoro-2-oxindole.

5-fluoro-2-oxindole (Figure 1) with a 97% of purity was purchased in Sigma-Aldrich (St. Louis, MO, USA), dissolved in dimethylsulfoxide (1% in 0.9% of saline solution) and intraperitoneally administered in a final volume of 10 mL/kg, 1 h before testing, in conformity with a preceding study [8]. Morphine hydrochloride acquired from Alcaiber S.A. (Madrid, Spain) was dissolved in 0.9% saline solution and subplantarly administered, in a final volume of 30 μL, 30 min before doing the behavioral tests [11,19]. All drugs were freshly prepared before use. For each group treated with a drug, the respective control group received the same volume of corresponding vehicle.

### 2.8. Statistical Analyses

All data are expressed as the mean values ± standard error of the mean (SEM). We used the SPSS program (version 13 for Windows, IBM, Madrid, Spain) for the statistical analysis. A three-way repeated-measures ANOVA with paw, treatment and time as the factors of variation with the corresponding one-way ANOVA and Student Newman Keuls test was used to analyze the effects of 5-fluoro-2-oxindole on nociception. A one-way ANOVA followed by the Student Newman Keuls test was utilized for evaluating the effects of 5-fluoro-2-oxindole combined with SnPP or morphine. The effects of 5-fluoro-2-oxindole in the expression of several proteins were analyzed using a one-way ANOVA and the Student Newman Keuls test. A value of *p* < 0.05 was considered significant.

In the von Frey filaments and plantar tests, antinociception is expressed as the percentage of maximal possible effect, where the test latencies pre-drug (baseline) and post-drug administration are compared and calculated in accordance with this equation

Maximal possible effect (%) = [(drug-baseline)/(cut-off-baseline)] × 100

## 3. Results

### 3.1. The Antinociceptive Effects of 5-Fluoro-2-Oxindole during Peripheral Inflammation

**Figure 2:**
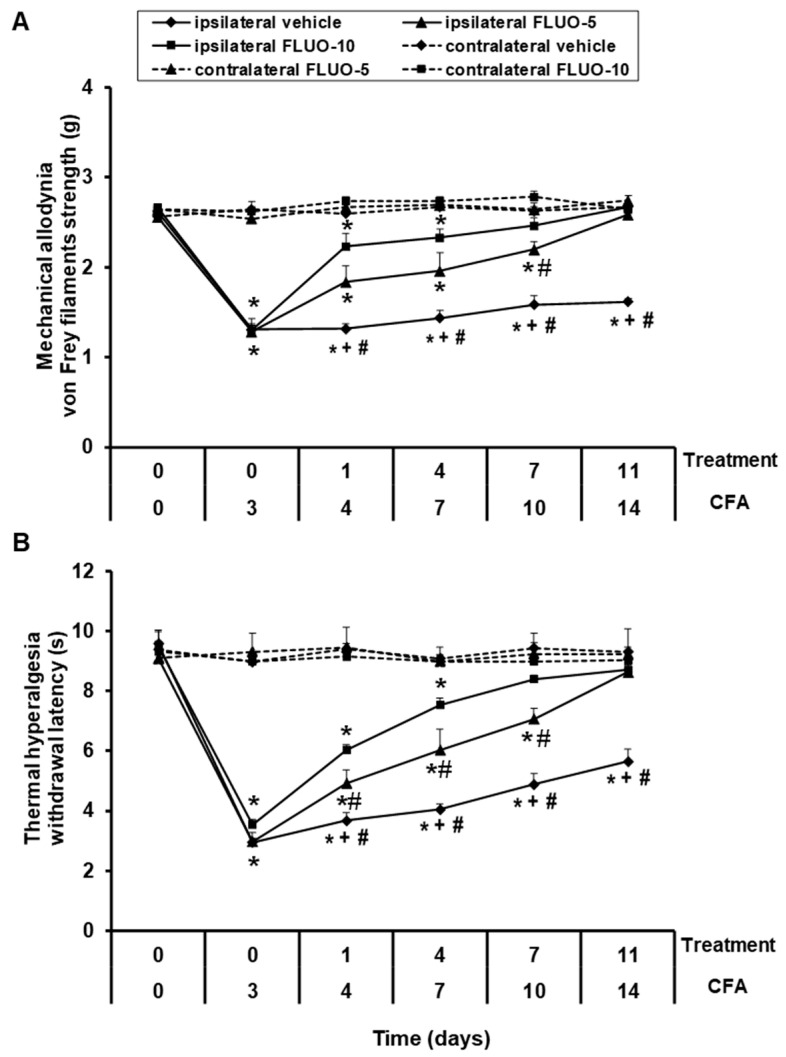
Treatment with 5-fluoro-2-oxindole decreases the mechanical allodynia and thermal hyperalgesia induced by CFA. The development of (**A**) mechanical allodynia and (**B**) thermal hyperalgesia in both hind paws of CFA-injected mice treated with 5-fluoro-2-oxindole (FLUO) or vehicle for 11 consecutive days are shown. The effects of 5 and 10 mg/kg 5-fluoro-2-oxindole were evaluated at days 4, 7, 10 and 14 after CFA injection. For each day and treatment evaluated, * indicates significant differences vs. their respective contralateral paws, + indicates significant differences vs. ipsilateral paws of animals treated with FLUO at 5 mg/kg, and # indicates significant differences vs. ipsilateral paws of animals treated with FLUO at 10 mg/kg (*p <* 0.05, one-way ANOVA followed by Student-Newman-Keuls test). Results are represented as mean ± S.E.M. values; *n* = 6 animals per experimental group.

Data showed that the repetitive administration of 5-fluoro-2-oxindole reduced the allodynia (Figure 2A). Significant effects of paw (*p <* 0.001), treatment (*p <* 0.001) and time (*p <* 0.001), and interactions between paw × treatment (*p <* 0.001), paw × time (*p <* 0.011), treatment × time (*p <* 0.001) as well as among paw × treatment × time (*p <* 0.001) were revealed by the three-way repeated-measures ANOVA. A gradual enhancement of the ipsilateral hind paw withdrawal threshold in response to von Frey filaments since days 1 to 11 of 5-fluoro-2-oxindole treatment was demonstrated (*p <* 0.001; one-way ANOVA vs. the respective ipsilateral paws of mice treated with vehicle). Treatment with 5-fluoro-2-oxindole at 10 mg/kg completely inhibited the allodynia induced by peripheral inflammation after 7 days of treatment, while 11 days of treatment are required to block the allodynia with the administration of 5 mg/kg of this drug.

For thermal hyperalgesia, the three-way repeated measures ANOVA also revealed significant effects of paw, treatment and time (*p <* 0.001), and interactions among paw × treatment, paw × time, treatment × time and paw × treatment × time (*p <* 0.001). Treatment with 5-fluoro-2-oxindole also exhibited a progressive enhance in the latency of paw withdrawal since day 1 to day 11 of treatment (*p <* 0.001; one-way ANOVA and Student Newman Keuls test vs. the ipsilateral paws of mice treated with vehicle, Figure 2B). The hyperalgesia caused by peripheral inflammation was totally blocked at 7 and 11 days of the repetitive administration with 10 and 5 mg/kg of 5-fluoro-2-oxindole, respectively.

In both tests, the intraperitoneal administration of 5 or 10 mg/kg of 5-fluoro-2-oxindole did not have any action in the contralateral paws of animals with peripheral inflammation (Figure 2A,B).

### 3.2. Reversion of the Antinocicetptive Effects of 5-Fluoro-2-Oxindole with SnPP

**Figure 3:**
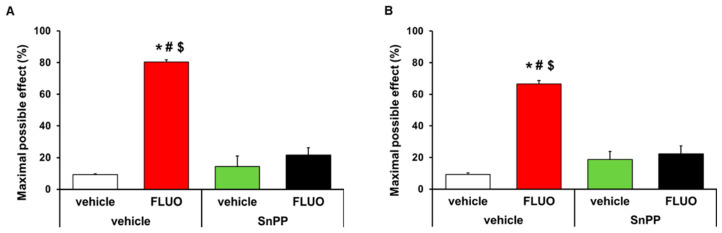
Reversion of the antinociceptive effects of 5-fluoro-2-oxindole by SnPP treatment. Effects of treatment with 5 mg/kg 5-fluoro-2-oxindole (FLUO) or vehicle during 11 consecutive days in the ipsilateral paw of mice co-treated with 5 mg/kg of SnPP or vehicle at day 14 after CFA injection in the inhibition of the mechanical allodynia (**A**) and thermal hyperalgesia (**B**). In both panels, * indicates significant differences vs. vehicle plus vehicle-treated mice, # indicates significant differences vs. SnPP plus vehicle-treated mice and $ indicates significant differences vs. SnPP plus FLUO treated mice (*p <* 0.05, one-way ANOVA followed by Student-Newman-Keuls test). Data are expressed as mean values of the maximal possible effect (%) ± S.E.M.; *n* = 6 animals per experimental group.

The antinociceptive effects generated by the intraperitoneal administration of 5 mg/kg 5-fluoro-2-oxindole during 11 consecutive days were inhibited with the intraperitoneal administration of 5 mg/kg SnPP. That is, the antiallodynic (*p <* 0.001, one-way ANOVA; Figure 3A) and antihyperalgesic (*p <* 0.001, one-way ANOVA; Figure 3B) effects induced by 5-fluoro-2-oxindole were completely inhibited with its co-administration with the HO-1 inhibitor. The administration of SnPP alone or combined with 5-fluoro-2-oxindole did not have any effect on the contralateral paws of CFA-injected animals (data not shown).

### 3.3. Effect of 5-Fluoro-2-Oxindole in the Expression of MAPK in the Spinal Cords and Paws of Mice Inflammatory Pain

**Figure 4:**
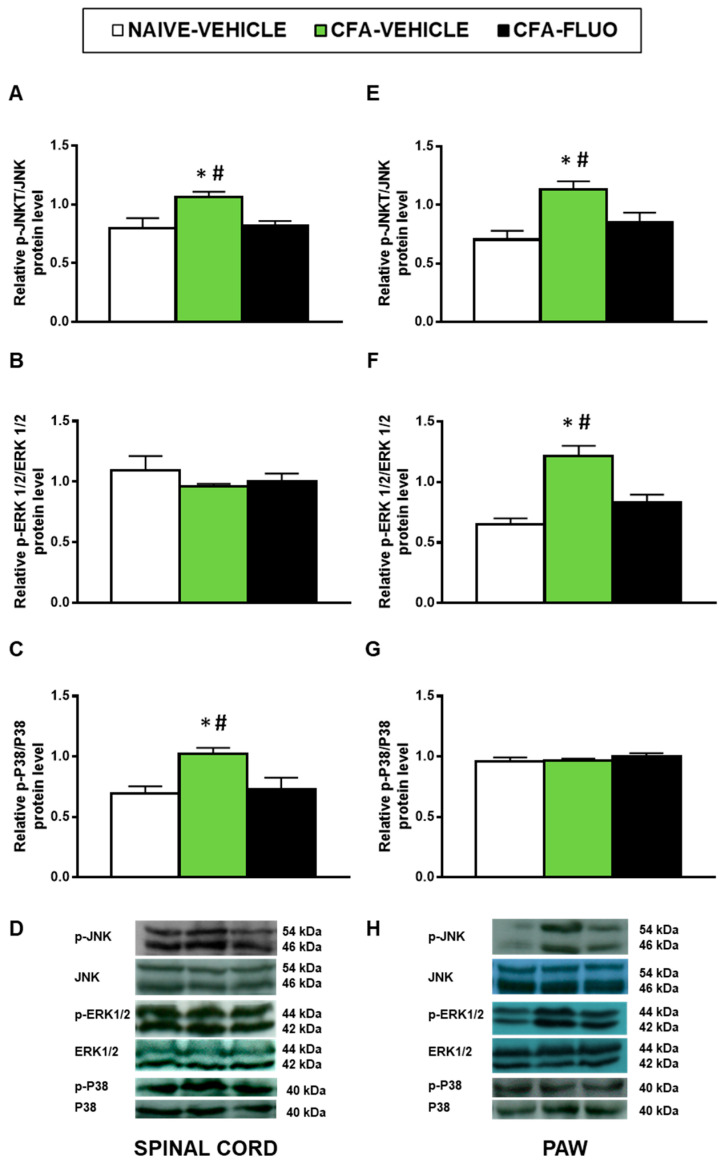
Treatment with 5-fluoro-2-oxindole normalized the activation of JNK, ERK 1/2 and P38 induced by CFA in spinal cords and/or paws. The relative protein levels of p-JNK/JNK (**A**,**E**), p-ERK 1/2/ERK 1/2 (**B**,**F**) and p-P38/P38 (**C**,**G**) in the spinal cords and paws of CFA-injected mice treated with 5 mg/kg 5-fluoro-2-oxindole (FLUO) or vehicle during 11 consecutive days are represented. We used naive vehicle treated animals as controls. Representative blots for p-JNK/JNK (46/54 kDa), p-ERK 1/2/ERK 1/2 (44/42 kDa) and p-P38/P38 (40 kDa) in the spinal cords (**D**) and paws (**H**) of animals with peripheral inflammation are shown. In all figures, * symbolizes significant differences compared with naïve vehicle treated mice and # vs. CFA-injected mice treated with 5-fluoro-2-oxindole (*p <* 0.05; one-way ANOVA and Student–Newman–Keuls test). The results are represented as the mean ± SEM; *n* = 3–4 samples per group.

Our findings demonstrated that CFA injection augmented the spinal cord expression of p-JNK (*p <* 0.029; one-way ANOVA; Figure 4A) and p-P38 (*p <* 0.016, one-way ANOVA; Figure 4C), and the paw levels of p-JNK (*p <* 0.018, one-way ANOVA; Figure 4E) and p-ERK 1/2 (*p <* 0.003, one-way ANOVA; Figure 4F). Moreover, the enhanced expression of p-JNK, p-ERK 1/2 and p-P38 was normalized by 5-fluoro-2-oxindole. Non-changes in the spinal cord levels of p-ERK 1/2 or in the paw levels of p-P38 were observed (Figure 4B,G).

### 3.4. Effects of 5-Fluoro-2-Oxindole in the Expression of Antioxidant Proteins in the Spinal Cords and Paws of Animals with Peripheral Inflammation

**Figure 5:**
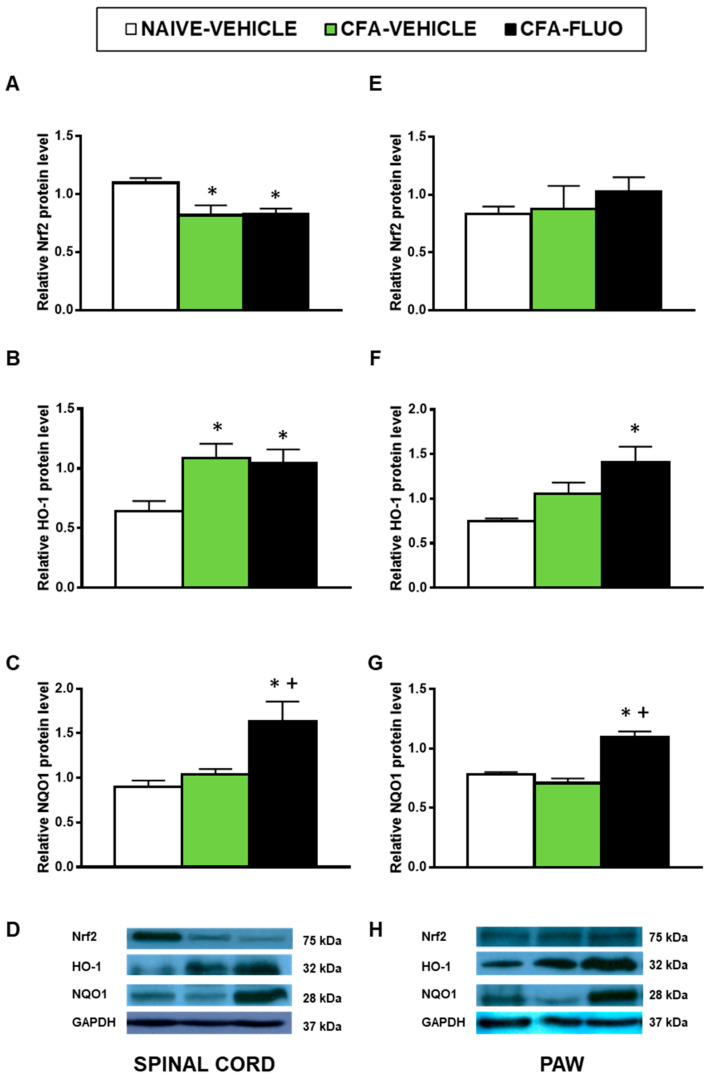
Treatment with 5-fluoro-2-oxindole increases the expression of HO-1 and NQO1 in the paws and/or spinal cords of mice with inflammatory pain. The relative protein levels of (**A**,**E**) Nrf2, (**B**,**F**) HO-1 and (**C**,**G**) NQO1 in the spinal cords and paws of CFA-injected mice treated with 5 mg/kg 5-fluoro-2-oxindole (FLUO) or vehicle during 11 consecutive days are presented. Naive mice treated with vehicle were used as controls. Representative blots for Nrf2 (75 kDa), HO-1 (32 kDa), NQO1 (28 kDa) and GAPDH (37 kDa) in the spinal cords (**D**) and paws (**H**) are shown. All proteins are expressed relative to GAPDH levels. In all panels, * denotes significant differences vs. naïve mice treated with vehicle and + vs. CFA-injected mice treated with vehicle (*p <* 0.05; one-way ANOVA and Student-Newman-Keuls test). The results are presented as the mean ± SEM; *n* = 3–4 samples per experimental group.

The injection of CFA decreased the spinal cord expression of Nrf2 (*p <* 0.016, one-way ANOVA; Figure 5A), and increased the spinal cord protein levels of HO-1 (*p <* 0.031, one-way ANOVA; Figure 5B). Non-changes caused by CFA were manifested in the Nrf2 (Figure 5E) and HO-1 (Figure 5F) levels in the paw, nor in the NQO1 levels in the spinal cords (Figure 5C) or paws (Figure 5G). The administration of 5-fluoro-2-oxindole did not amend the decreased protein levels of Nrf2 but maintained or increased the expression of HO-1 and NQO1 in the spinal cords and paws.

### 3.5. Effect of 5-Fluoro-2-Oxindole in the Protien Levels of 4-HNE, NOS2, CD11b/c, IBA-1 and MOR in the Spinal Cords and/or Paws of Animals with Peripheral Inflammation

**Figure 6:**
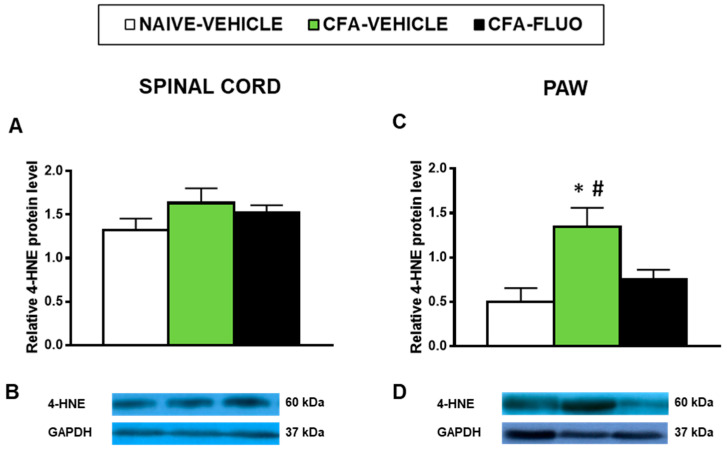
Treatment with 5-fluoro-2-oxindole inhibited the increased levels of 4-HNE-positive proteins in the paws of mice with inflammatory pain. The relative levels of 4-HNE-positive proteins in the spinal cords (**A**) and paws (**C**) of CFA-injected mice treated with 5 mg/kg 5-fluoro-2-oxindole (FLUO) or vehicle during 11 consecutive days are presented. Naive mice treated with vehicle were used as controls. Representative blots for 4-HNE-positive proteins (60 kDa) and GAPDH (37 kDa) in the spinal cords (**B**) and paws (**D**) are shown. In all panels, * denotes significant differences vs. naïve mice treated with vehicle and # vs. CFA-injected mice treated with FLUO (*p <* 0.05; one-way ANOVA and Student-Newman-Keuls test). The results are presented as the mean ± SEM; *n* = 3–4 samples per experimental group.

4-HNE is an oxidative stress marker with several positive bands in the Western blot; we selected the 60 kDa band to quantify by densitometry as it showed reaction in all samples and can provide a better parameter to compare changes in the levels of 4-HNE-positive proteins among all samples analyzed. Our results showed that the administration of CFA did not alter the levels of 4-HNE-positive proteins in the spinal cords (Figure 6A), but significantly increased its protein levels in the paws (*p <* 0.027, one-way ANOVA; Figure 6C), which were completely normalized by 5-fluoro-2-oxindole treatment.

**Figure 7:**
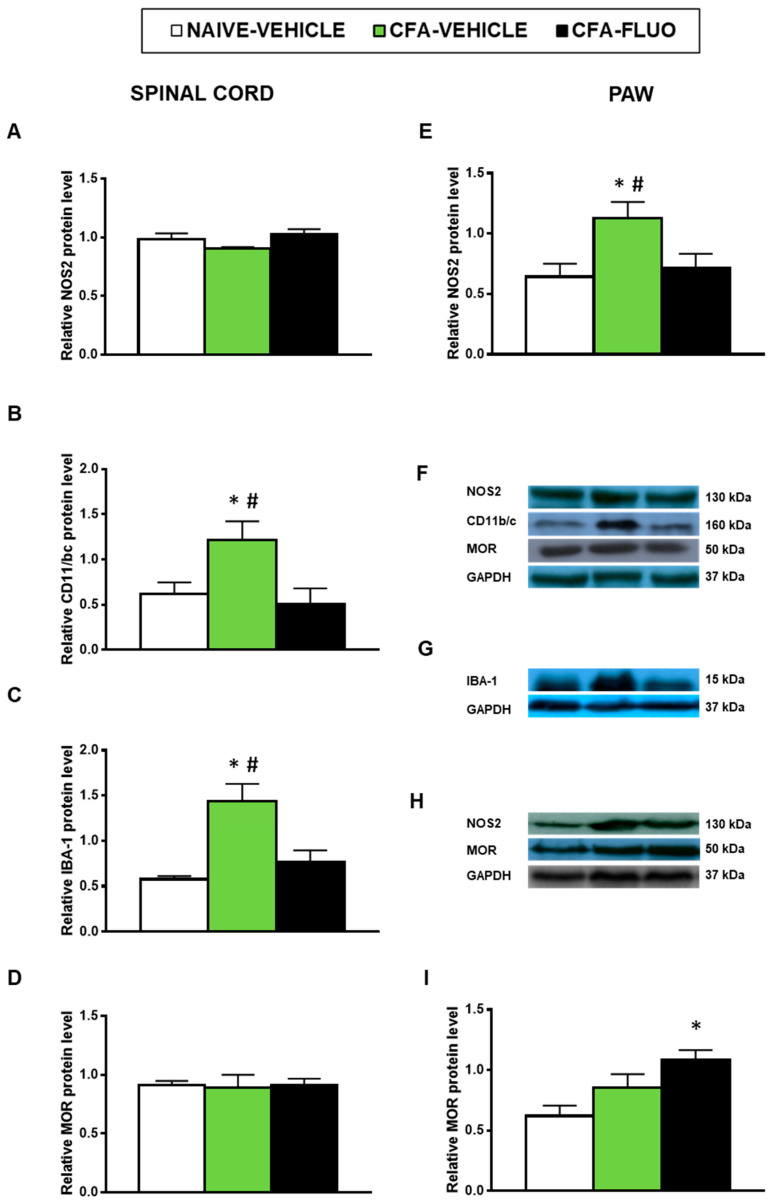
Treatment with 5-fluoro-2-oxindole normalized the paw NOS2 up-regulation and the spinal cord microglial activation and increased the paw expression of MOR in mice with inflammatory pain. The relative protein levels of (**A**,**E**) NOS2, (**B**) CD11b/c, (**C**) IBA-1, and (**D**,**I**) MOR in the spinal cords and/or paws of CFA-injected mice treated with 5 mg/kg 5-fluoro-2-oxindole (FLUO) or vehicle during 11 consecutive days are presented. Naive mice treated with vehicle were used as controls. (**F**) Representative blots for NOS2 (130 kDa), CD11b/c (160 kDa), MOR (50 kDa) and GAPDH (37 kDa) in the spinal cords are shown. (**G**) Representative blots for IBA-1 (15 kDa) and GAPDH (37 kDa) in the spinal cords are displayed. (**H**) Representative blots for NOS2 (130 kDa), MOR (50 kDa) and GAPDH (37 kDa) in the paws are shown. All proteins are expressed relative to GAPDH levels. In all panels, * denotes significant differences vs. naïve mice treated with vehicle and # vs. CFA-injected mice treated with 5-fluoro-2-oxindole (*p <* 0.05; one-way ANOVA and Student–Newman–Keuls test). The results are presented as the mean ± SEM; *n* = 3–4 samples per experimental group.

Our results also showed that the injection of CFA increased the spinal cord expression of CD11b/c (*p <* 0.047, one-way ANOVA; Figure 7B) and IBA-1 (*p <* 0.009, one-way ANOVA; Figure 7C) as well as the paw levels of NOS2 (*p <* 0.041, one-way ANOVA; Figure 7E). Non-alterations in the spinal cord NOS2 expression (Figure 7A) or in the protein levels of MOR in the spinal cords (Figure 7D) or paws (Figure 7I) were provoked by CFA. Treatment with 5-fluoro-2-oxindole normalized the up-regulation of NOS2 (Figure 7E) and those of CD11b/c (Figure 7B) and IBA-1 (Figure 7C). Our results further demonstrated that 5-fluoro-2-oxindole increased the paw levels of MOR (*p <* 0.023, one-way ANOVA; Figure 7I).

### 3.6. Treatment with 5-Fluoro-2-Oxindole Potencites the Local Antinociceptive Effects of Morphine

**Figure 8:**
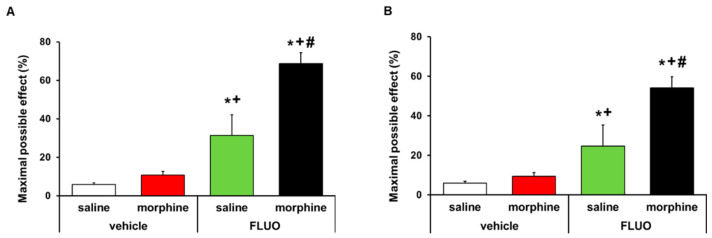
Treatment with 5-fluoro-2-oxindole enhanced the antiallodynic and antihyperalgesic effects of morphine. Effects of the acute administration of 5 mg/kg 5-fluoro-2-oxindole (FLUO) or vehicle in combination with 50 µg of morphine or saline in the inhibition of the mechanical allodynia (**A**) and thermal hyperalgesia (**B**) induced by CFA in the ipsilateral paws. In all panels, * indicates significant differences vs. vehicle plus saline-treated mice, + indicates significant differences vs. vehicle plus morphine-treated mice, and # indicates significant differences vs. FLUO plus saline-treated mice (*p <* 0.05, one-way ANOVA followed by Student–Newman–Keuls test). Data are expressed as mean values of the maximal possible effect (%) ± S.E.M.; *n* = 6 animals per experimental group.

The effects of the acute intraperitoneal administration of 5 mg/kg 5-fluoro-2-oxindole alone and combined with 50 μg morphine subplantarly injected in the allodynia and hyperalgesia provoked by peripheral inflammation were assessed. Data revealed that 5-fluoro-2-oxindole significantly enhanced the antiallodynic (Figure 8A) and antihyperalgesic effects (Figure 8B) of morphine in animals with inflammatory pain (*p <* 0.001, one-way ANOVA, vs. their respective vehicle groups treated with saline or morphine and vs. groups treated with 5-fluoro-2-oxindole plus saline). Morphine administered alone or combined with 5-fluoro-2-oxindole did not have any effect in the contralateral paws of CFA-injected animals (data not shown).

## 4. Discussion

This study reveals that the repeated administration of 5-fluoro-2-oxindole inhibited the mechanical allodynia and thermal hyperalgesia induced by CFA, whose effects were reversed with the administration of SnPP (a HO-1 inhibitor). Treatment with 5-fluoro-2-oxindole also inhibited MAPK phosphorylation, potentiated the expression of the antioxidant enzymes HO-1 and NQO1, normalized the CFA-induced oxidative stress, NOS2 overexpression and microglial activation. This drug also enhanced the local expression and the antinociceptive effects of MOR.

The role of oxindoles in the modulation of chronic inflammatory pain has not been widely studied. Previous studies demonstrated that the administration of several oxindoles such as convolutamydine A (4,6-bromo-3-(2-oxopropyl)-3-hydroxy-2-oxindole), 3-(2-oxopropyl)-3-hydroxy-2-oxindole and 5-bromo-3-(2-oxopropyl)-3-hydroxy-2-oxindole inhibited visceral and acute inflammatory pain [5,6,7]. A recent work evidenced the antinociceptive effects of 5-fluoro-2-oxindole in animals with chronic neuropathic pain [8]. Even so, the likely analgesic effects of this oxindole during chronic inflammatory pain are unknown. Our results showed, for the first time, that the administration of 5 and 10 mg/kg 5-fluoro-2-oxindole inhibited the mechanical allodynia and thermal hyperalgesia induced by peripheral inflammation in a different effectiveness. Indeed, whereas seven days of treatment with 10 mg/kg 5-fluoro-2-oxindole completely blocked the mechanical allodynia and thermal hyperalgesia generated by CFA, eleven days of treatment with 5 mg/kg of this drug are required to completely reverse the allodynia and hyperalgesia. These results agree with the dose-response inhibitory effects of 5-fluoro-2-oxindole in animals with neuropathic pain [8], as well as with the effects of other oxindoles in different murine models of acute inflammatory pain [5,6].

In this study, we further demonstrated that 5-fluoro-2-oxindole inhibited the activation of MAPK induced by peripheral inflammation. The activation of JNK has an important role in the development and maintenance of chronic pain [24,25]. In accordance with Gao et al. [26], our findings demonstrated increased levels of phosphorylated JNK in the spinal cord and paw of mice with chronic inflammatory pain and revealed that treatment with 5-fluoro-2-oxindole completely reduced the JNK activation in both tissues. Since the administration of specific JNK inhibitors inhibited the mechanical allodynia caused by inflammation [26], the decreased activation of JNK performed by 5-fluoro-2-oxindole suggested the possible involvement of this MAPK in the mechanism of action of this oxindole under inflammatory pain conditions. ERK 1/2 is another MAPK whose phosphorylated form also increased in response to several stimuli, for example, inflammation, nerve injury or diabetes [27,28]. The increased expression of p-ERK 1/2 detected in the paw of CFA-injected mice was also inhibited by 5-fluoro-2-oxindole. Moreover, P38 activation also mediates the hyperalgesia-induced by peripheral inflammation [29] and the overexpression of p-P38 detected in the spinal cord of CFA-injected mice was inhibited by 5-fluoro-2-oxindole treatment, suggesting that the effectivity of this treatment during chronic inflammatory pain also comprises the inhibition of ERK 1/2 and P38 phosphorylation in the paw and spinal cord, respectively. These results are in agreement with the inhibition of MAPK phosphorylation caused by IRN and RIN in LPS-stimulated microglial cells [12].

Several studies showed microglial activation in the spinal cord of animals with inflammatory pain [30], and the increased expression of CD11b/c and IBA-1 observed in the spinal cord of our animals supported these findings. Moreover, and in accordance with the inhibition of activated microglia made by 5-fluoro-2-oxindole in sciatic nerve-injured mice [8], this treatment also blocked the spinal high levels of CD11b/c and IBA-1 induced by CFA-injection, revealing that the antinociceptive activities of this drug might also be produced via microglial inactivation. The activated microglia mediate the release of several inflammatory proteins, for example, TNFα, and interleukins, and its inhibition reduced the allodynia and hyperalgesia in animals with chronic pain [31]. Nitric oxide also contributes to inflammatory pain induction [11,20] and the high paw levels of NOS2 induced by CFA were inhibited by 5-fluoro-2-oxindole. These data agree with the suppression of NOS2 overexpression caused by IRN and/or RIN in LPS-stimulated murine microglial cells [12] and alveolar macrophages [3] as well as with those produced by Nrf2 inducers in the paw of CFA-injected mice [11], thus revealing the modulatory role played by 5-fluoro-2-oxindole in the synthesis of nitric oxide mediated by NOS2. Considering the potent analgesic actions of several specific NOS2 inhibitors during peripheral inflammation [32], the analgesic effects of 5-fluoro-2-oxindole might also be mediated by inhibiting the NOS2 up-regulation.

Numerous works demonstrated the inhibitory effects induced by the Nrf2, HO-1 and NQO1 signaling pathway activation in inflammatory pain. Thus, different analgesics for example sulforaphane and CORM’s mediated their antinociceptive effects by activating the expression of HO-1 and NQO1 in animals with inflammatory [11,33] or neuropathic pain associated with type 1 and 2 diabetes [34,35,36] and caused by nerve injury [14,18]. In this work, we demonstrated that treatment with 5-fluoro-2-oxindole increased the expression of NOQ1 and maintained or enhanced the high protein levels of HO-1 induced by peripheral inflammation in the spinal cords and paws, but did not alter the decreased spinal cord expression of Nrf2 provoked by peripheral inflammation, indicating that this treatment acts directly on the downstream pathway activated by this transcription factor. In accordance with our findings, 5-fluoro-2-oxindole also enhanced the expression of HO-1 and NQO1 in the spinal cord and/or hippocampus of sciatic nerve-injured animals [8]. The augmented expression of HO-1 stimulated by peripheral inflammation in the spinal cord are in conformity with the overexpression of this enzyme detected in the dorsal root ganglia of these animals [37], suggesting that the high expression of this antioxidant protein might act as a defense mechanism against the oxidative stress triggered by paw inflammation. Nevertheless, the maintenance of the high spinal cord levels of HO-1 and its increased expression in the paw of 5-fluoro-2-oxindole treated animals, together with the reversion of the antinociceptive effects of 5-fluoro-2-oxindole by SnPP (a HO-1 inhibitor), sustain the possibility that the antinociceptive effects induced by this oxindole during inflammation was produced by preserving and/or potentiating the activation of this antioxidant enzyme. Our data also showed that the expression of NQO1 was significantly increased in the spinal cords and paws of 5-fluoro-2-oxindole treated mice. This enzyme also contributes to the inhibition of diabetic neuropathy made by Nrf2 and HO-1 activators in animals with type 2 diabetes [35,36] and plays a relevant neuroprotective role in several physical and mental disorders by neutralizing the reactive oxidative species (ROS) [38,39]. Several works demonstrated that ROS are generated at the site of inflammation and one major action of ROS is the production of 4-HNE among to other reactive carbonyl species [40,41,42]. Our results support these finding by demonstrating increased levels of 4-HNE-positive proteins in the paw of CFA-injected animals showing the oxidative stress induced by peripheral inflammation. Considering that the paw injection of 4-HNE induced mechanical hypersensitivity [40,41] and treatment with 5-fluoro-2-oxindole inhibited the high levels of 4-HNE-positive proteins in the paw of CFA-injected mice, we propose that 5-fluoro-2-oxindole inhibits inflammatory pain by attenuating oxidative stress. Moreover, and taking into account that oxidative stress is directly linked to the HO-1/NQO1 signaling pathway, the potentiation of these antioxidant enzymes and the inhibition of 4-HNE-positive proteins induced by 5-fluoro-2-oxindole further support that the antioxidant effects of this treatment are also involved in their analgesic actions during inflammatory pain.

**Figure 9:**
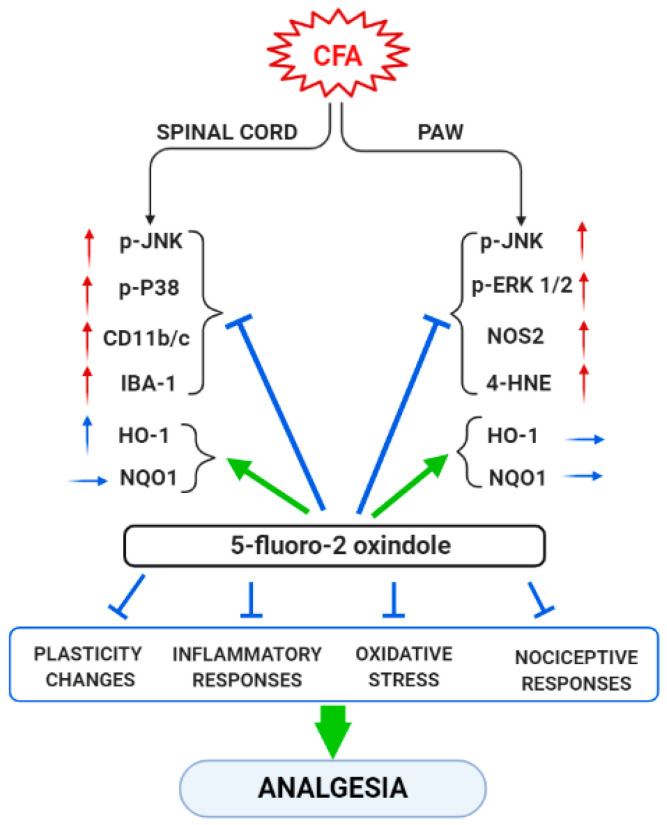
Summarizing scheme of the effects of treatment with 5-fluoro-2-oxindole in the spinal cord and paw of animals with CFA-induced inflammatory pain. ↑, indicates increase and → indicates no changes. Arrows in red and blue are related with pro-nociceptive and antinociceptive actions, respectively.

In summary, our data reveal that peripheral inflammation does not activate the same MAPK, inflammatory or oxidative stress markers in all tissues. Thus, while JNK is activated in the spinal cord and paw of CFA-injected animals, the other MAPK (ERK and p-38) were only activated in one of these tissues, paw and spinal cord, respectively. Moreover, whereas the expression of Nrf2 or HO-1 were decreased or increased in the spinal cord, non-changes in their protein levels were detected in the paws. In contrast, CFA-injection only upregulated the paw’s, but not the spinal cord’s, levels of 4-HNE-positive proteins and NOS2, while both microglial markers (CD11b/c and IBA-1) were up-regulated in the spinal cord of animal with peripheral inflammation. These results suggested that the plasticity, inflammatory and oxidative stress changes induced by CFA take place in both tissues, but they were differentially expressed according to the type of marker analyzed. Nonetheless, 5-fluoro-oxindole inhibited JNK and p-38 phosphorylation and microglial activation in the spinal cord as well as the phosphorylation of JNK and ERK 1/2 and the increased levels of 4-HNE-positive proteins and NOS2 in the paw. The protein levels of HO-1 and NQO1 were also maintained at high or augmented levels after 5-fluoro-oxindole treatment in both tissues. These results show the effects of 5-fluoro-2-oxindole in the spinal cords and paws of animals with inflammatory pain and provide new mechanisms of action of this oxindole under inflammatory pain conditions (Figure 9).

This work has some limitations: (1) the lack of histological studies evaluating the effects of 5-fluoro-2 oxindole on the morphological changes induced by CFA, (2) that we only determined total Nrf2 expression instead of expression ratio of cytosolic and nuclear Nrf2, that would allow a more accurate evaluation of the effects of inflammation and 5-fluoro-2 oxindole in the expression of Nrf2 and (3) we only quantified one 4-HNE-positive protein band among several.

Our results further reported that 5-fluoro-2-oxindole increased the protein levels of MOR in the paws of CFA-injected mice. The fact that this treatment also activated the HO-1 synthesis and this enzyme incited the upregulation of peripheral MOR in mice with inflammatory pain [19], allows us to theorize that the HO-1 signaling pathway activation might be implicated in the MOR overexpression induced by 5-fluoro-2-oxindole. Finally, and considering the enhanced paw expression of MOR induced by this drug, we evaluated the effects of this treatment on the antinociceptive actions produced by the local administration of morphine. Our data revealed that the antinociceptive effects of morphine were significantly enhanced by 5-fluoro-2-oxindole co-treatment. Likewise, the antiallodynic and antihyperalgesic effects of morphine in 5-fluoro-2-oxindole pre-treated animals were enhanced by 57.9% and 44.7% as compared with the effects of morphine administered alone. These results agree with the potentiation of the antinociceptive effects of morphine induced by this treatment during neuropathic pain [8], as well as with the improvement in the peripheral antinociceptive actions of morphine generated by CoPP in animals with inflammatory and neuropathic pain [18,19,43], and further proposed a new approach to potentiate the local effects of opioids as an alternative for chronic inflammatory pain treatment.

## 5. Conclusions

In summary, this study reports that 5-fluoro-2-oxindole alleviates inflammatory pain and improves the analgesic effects of morphine. This treatment inhibits the plasticity changes, oxidative stress and inflammatory responses caused by peripheral inflammation and increases the local expression of MOR. This work reveals that the administration of 5-fluoro-2-oxindole alone and/or combined with morphine are two remarkable new procedures for treating chronic inflammatory pain.
